# Baseline assessment of patient safety culture in primary care centres in Kuwait: a national cross-sectional study

**DOI:** 10.1186/s12913-021-07199-1

**Published:** 2021-10-28

**Authors:** Talal ALFadhalah, Buthaina Al Mudaf, Hanaa A. Alghanim, Gheed Al Salem, Dina Ali, Hythem M. Abdelwahab, Hossam Elamir

**Affiliations:** 1 Quality and Accreditation Directorate, Ministry of Health, Kuwait City, Kuwait; 2 Assistant Undersecretary of Public Health Affairs, Ministry of Health, Kuwait City, Kuwait; 3 Safety Department, Quality and Accreditation Directorate, Ministry of Health, Kuwait City, Kuwait; 4 Accreditation Affairs Department, Quality and Accreditation Directorate, Ministry of Health, Kuwait City, Kuwait; 5 National Blood Transfusion Services, Ministry of Health and Population, Giza, Egypt; 6 Research and Technical Support Department, Quality and Accreditation Directorate, Ministry of Health, Kuwait City, Kuwait

**Keywords:** Kuwait, Primary care, Patient safety, MOSPSC, Safety culture

## Abstract

**Background:**

Assessments of the culture surrounding patient safety can inform healthcare settings on how their structures and processes impact patient outcomes. This study investigated patient safety culture in Primary Health Care Centres in Kuwait, and benchmarked the findings against regional and international results. This study also examined the association between predictors and outcomes of patient safety culture in these settings.

**Methods:**

This cross-sectional quantitative study used the Medical Office Survey on Patient Safety Culture. The study was targeted at staff of all the Primary Health Care Centres in Kuwait with at least one year of experience. Data were analysed using SPSS 23 at a significance level of ≤ .05. Univariate (means, standard deviations, frequencies, percentages) and bivariate (chi-squared tests, student *t*-tests, ANOVA F-tests, Kruskal–Wallis tests, Spearman’s correlation) analyses provided an overview of participant socio-demographics and the association between patient safety culture composites and outcomes. We undertook a multivariate regression analysis to predict the determinants of patient safety culture. Results were benchmarked against similar local (Kuwait, 2014), regional (Yemen, 2015) and international (US, 2018) studies.

**Results:**

The responses of 6602 employees from 94 centres were included in the study, with an overall response rate of 78.7%. The survey revealed Teamwork (87.8% positive ratings) and Organisational Learning (78.8%) as perceived areas of strength. Communication about Error (57.7%), Overall Perceptions of Patient Safety and Quality (57.4%), Communication Openness (54.4%), Owner/Managing Partner/Leadership Support for Patient Safety (53.8%) and Work Pressure and Pace (28.4%) were identified as areas requiring improvement. Benchmarking analysis revealed that Kuwait centres are performing at benchmark levels or better on four and six composites when compared to international and regional findings, respectively. Regression modelling highlighted significant predictions regarding patient safety outcomes and composites.

**Conclusions:**

This is the first major study addressing the culture of patient safety in public Primary Health Care Centres regionally. Improving patient safety culture is critical for these centres to improve the quality and safety of the healthcare services they provide. The findings of this study can guide country-level strategies to develop the systems that govern patient safety practices.

**Supplementary Information:**

The online version contains supplementary material available at 10.1186/s12913-021-07199-1.

## Background

Patient safety is a central indicator of quality in healthcare, and has emerged as a significant field of study [1–4]. It is a discipline that applies scientific methods of safety to produce a reliable system that reduces the occurrence and harm of adverse events [5]*.* Even two decades after the landmark report *To Err is Human* [6] was published, the complexities of healthcare systems and uncoordinated efforts to address safety concerns ensure that the incidence of adverse events remains unacceptably high [2, 4, 7].

Improving patient safety requires the culture surrounding it to be assessed, and doing so helps an organisation identify the areas requiring improvement and to track changes over time [1, 8, 9]. Workplace culture is created by the staff’s shared basic assumptions, values, attitudes and behaviours that differentiate an organisation from others [10, 11]. Based on that general understanding, safety culture is defined as the product of those attributes in regards to daily safety issues that interact with an organisation’s structure and determine that organisation’s health and safety management [1, 7, 12, 13]. In their quest to study safety culture, researchers have used quantitative, qualitative and mixed approaches to understand the challenges facing healthcare organisations in providing safe care [14–16]. They identified leadership support, teamwork, communication and feedback, and workload and burnout as common themes that impact safety culture [16–18]. Systematic reviews have revealed that participants from primary or secondary care settings rate teamwork among the highest positive dimensions and non-punitive response to errors as an area of concern [1, 19].

Kuwait is a high-income country with healthcare services provided mainly by a public system owned, funded, solely regulated, and operated by the Ministry of Health (MOH) [20–22]. Five health regions manage the different care levels. Primary care is provided at Primary Health Care Centres (PHCs), whereas hospitals provide secondary, tertiary and more specialised care [21]. More than 100 PHCs cover the country, a number that will grow with the building of new primary care clinics in a series of Public–Private Partnership projects [23].

Media reports on the failures of the healthcare system prompted the Kuwaiti Parliament and the public to demand improvements [12]. In response to growing pressures, the MOH initiated a research project aimed at assessing and improving patient safety [12, 24]. That project began with assessing patient safety culture (PSC) in secondary and tertiary care settings, that is, government hospitals. In line with international studies, this nationwide hospital study rated teamwork as an area of strength and non-punitive response to error as the worst aspect [24]. Smaller studies from Kuwait have reported the same findings [25, 26].

The present study aims at exploring PSC in public primary care settings in Kuwait, as reported by healthcare providers. The main objective of this study is to assess PSC in PHCs in Kuwait, and in particular, identify cultural strengths and weaknesses, benchmark results against regional and international studies, examine the socio-demographic determinants of the PSC, and explore the association between culture predictors and outcomes.

## Methods

### Study design and setting

We adopted a cross-sectional research design. To be representative of the national public primary care sector, the study was conducted in 100 PHCs grouped into the five health regions.

### Research tool

We circulated a self-administered questionnaire, the Medical Office Survey on Patient Safety Culture (MOSPSC). It is a validated and reliable tool developed by the Agency for Healthcare Research and Quality (AHRQ). The AHRQ reported that the internal consistency reliability statistics based on pilot test data from 202 medical offices and more than 4200 staff showed sound psychometric properties [27]. The reliability (Cronbach’s *α*) of the composites ranges from .75 for Communication about Error to .83 for Teamwork.

Asking about various aspects of PSC at primary health care settings, the original survey includes 38 items grouped into 10 composites. These composites are Teamwork, Patient Care Tracking/Follow-up, Organisational Learning, Overall Perceptions of Patient Safety and Quality, Staff Training, Owner/Managing Partner/Leadership Support for Patient Safety, Communication about Error, Communication Openness, Office Processes and Standardisation, and Work Pressure and Pace. The survey also asked about problems in exchanging information with other settings and about access to care. Participants were asked to rate the degree to which their PHC is patient-centred, effective, timely, efficient and equitable, and give an overall rating on patient safety. We refer to these sections as “patient safety and quality outcomes”.

The original survey is divided into nine sections (A to I), the last of which covers the participant’s background. The tool was pilot tested and modified for the PHC setting to minimise potential technical/language/cultural issues. We introduced some modifications to account for the differences between the primary care settings in Kuwait and the medical offices in the US. As we have larger facilities in Kuwait with more staff and specialities, we inserted a five-item section (new Section B) to assess information exchange within a PHC. Similar to the original section (Section B in the original questionnaire, Section C in our version), which assesses the exchange of information with other settings (external laboratories and imaging centres, pharmacies and hospitals), our new section assesses internal information exchange between the primary care centre’s laboratories, imaging services, its pharmacy and other clinics/physicians. We also modified items for collecting background information (Section J) to reflect the different job titles/ranks and provide information necessary to assess the relationship between socio-demographics and safety culture scores and outcomes. The tool was translated into Arabic and tested for reliability to be able to compare international results with ours. Hard copies of English (Appendix 1) and Arabic (Appendix 2) language versions were utilised in the study according to the preference of the participant. The items were rated on five-point Likert scales for agreement or six-point scales for frequency.

### Sampling and data collection

We utilised a total population sampling technique (*n* = 8389). We included all primary care staff with at least one year of experience who are expected to have sufficient knowledge about their PHC and its operations to provide informed answers to the survey. This includes clinical, allied, administrative and managerial staff members. We excluded staff on administrative or extended sick leave, those who have moved to another health region/centre, and those with less than one year of experience at the centre.

Survey forms were distributed through points of contact at each PHC. Data collection took place in April and May 2018. Points of contact facilitated data collection and ensured a high response rate. The safety and risk management team in each of the five health regions supervised data collection activities. To guarantee a consistent application of the survey, we held a full-day methodology workshop to ensure a common and comprehensive understanding of the study protocol and the methods of data collection and communication.

### Data management and analysis

Once the data was collected in full, we checked a random sample (10%) for accuracy and completeness to ensure data quality and integrity. The Cronbach’s *α* of the 10 composites together was found to be .891, which indicates an acceptable degree of internal consistency. Participant identities, and their PHC and health region identities, were coded for anonymity. At least one section had to be completed for that returned questionnaire to be included in the analysis.

In accordance with the AHRQ’s MOSPSC user’s guide [28], positive ratings on the Likert scale (Strongly Agree/Agree or Excellent/Very Good) were combined. The same was done for negative ratings (Disagree/Strongly Disagree or Poor/Fair). The midpoints of scales (Neither, Sometimes or Good) were regarded as a separate category. Several items in the survey were negatively worded, all of which were reverse-coded before the percentage of positive ratings were calculated for each composite and categorisation was performed. Following the Yemen study of 2015, the scores for the average percentage of positive ratings were categorised as follows: composites with scores ≥75% were designated “areas of strength”, whereas “areas for improvement” were those scoring below 60% [9].

We benchmarked results from the participating PHCs against similar international [29] and regional [9] assessments. We also compared the results of this present study with those of another that was conducted in Kuwait [25] but used the Hospital Survey on Patient Safety Culture (HSOPSC), the hospital version of the AHRQ tool [30]. Comparisons to the benchmark results were made using the following formula [24]:

% Distance from benchmark = [(PHC result – benchmark result)/benchmark result] × 100.

Results greater than + 10% were categorised as exceeding the benchmark, whereas results within 10% were defined as meeting the benchmark. Those between − 10 and − 30% were categorised as deviating slightly from the benchmark, whereas those below − 30% were considered to deviate greatly from the benchmark.

Data were analysed using SPSS 23 (*p-*value ≤ .05). Internal consistency was calculated using Cronbach’s alpha. The Kolmogorov–Smirnov and Shapiro–Wilk tests were used to check the normality of the data. The analysis of the quantitative data included univariate descriptive (means, standard deviations, frequencies, percentages) and bivariate (chi-squared tests, student *t*-tests, ANOVA F-tests) analyses to examine the association between the PSC composites and the outcome variables, and to examine how trends in the outcome variables differ across PHC and respondent characteristics. Non-parametric tests (Kruskal–Wallis test, Spearman’s correlation) were used if violations of assumptions did not allow using parametric testing. In these analyses, we used the respondent’s scores on the Likert scale (1–5) instead of the percentages of positive ratings used in the benchmarking analysis. The analysis also included multivariate analysis (regression) to construct a simulation model that can help predict the determinants of PSC and develop actionable strategies. Independent variables included in the multiple regression models were those that were found to be statistically significant (*p* ≤ .05) in the correlational analysis, with a correlation coefficient ≥ 0.100. To obtain the best-fitting regression models, some variables were excluded. We included the statistically significant correlated socio-demographic factors to predict the 10 composite scores. We ran another group of multiple regression analyses to predict scores for patient safety and quality outcomes.

## Results

Of the 8389 questionnaires distributed at the 100 PHCs, 7392 (88.1%) were returned from 94. The returned questionnaires were screened for their eligibility, which resulted in the exclusion of a further 790, yielding a final response rate of 78.7% (*n* = 6602).

### Socio-demographics

Table 1 shows the numbers and percentages of socio-demographic characteristics. More than half of the responses came from PHCs in two health regions. Almost three-quarters of participants (72.9%) were working in PHCs with more than 100 staff. With respect to in-house support services, 76.2% of responses indicated that the PHC has a laboratory service. Of the responses to the question on type of PHC location, 82.2% indicated an urban location. With respect to gender, 73.0% of responses were females. Nurses were the largest group (31.9%) responding to the question on position within their PHC. On the question of nationality, the majority (53.4%) of respondents were non-Kuwaiti nationals. Of the participants that indicated their age, 64.3% were aged between 30 and 45 years old. In terms of education, the largest responding group were those holding a university degree (35.3%).

**Table 1:** Participants’ socio-demographics and means of “average patient safety culture percentage across all composites”

	Number	%	Mean (SD)	*p*
**Health Region**				< .001
1	1665	25.2	3.43 (0.39)	
2	1274	19.3	3.38 (0.41)	
3	1136	17.2	3.38 (0.42)	
4	765	11.6	3.47 (0.35)	
5	1762	26.7	3.45 (0.38)	
**PHC**				
Specialised vs non-specialised clinics				.006
Specialised	916	13.9	3.39 (0.38)	
Non-specialised	5686	86.1	3.43 (0.40)	
Size				< .001
< 51	352	5.3	3.54 (0.38)	
51–100	1440	21.8	3.42 (0.41)	
> 100	4810	72.9	3.41 (0.39)	
Clinical support services				.082
With lab	5034	76.2	3.42 (0.39)	
With lab & radiology	883	13.4	3.44 (0.41)	
Without lab & radiology	685	10.4	3.42 (0.41)	
Location				.002
Urban	5425	82.2	3.43 (0.40)	
Suburban	1149	17.4	3.39 (0.39)	
Rural	28	0.4	3.47 (0.40)	
**Participants**				
Gender				.511
Male	1684	27.0	3.43 (0.39)	
Female	4549	73.0	3.42 (0.39)	
Age				< .001
Below 30 years	1072	17.1	3.38 (0.43)	
30–45 years	4017	64.3	3.43 (0.39)	
46–55 years	810	13.0	3.42 (0.39)	
Over 55 years	353	5.6	3.48 (0.39)	
Language				< .001
Arabic	3989	69.7	3.38 (0.43)	
English	1736	30.3	3.52 (0.29)	
Nationality				< .001
Kuwaiti	2881	46.6	3.36 (0.43)	
Arabian	1471	23.8	3.43 (0.39)	
Asian	1482	24.0	3.53 (0.29)	
European/American	13	0.2	3.50 (0.45)	
Other	339	5.5	3.43 (0.39)	
Highest educational credential				< .001
Below high school	341	5.8	3.38 (0.38)	
High school	727	12.3	3.39 (0.40)	
Technical school	537	9.1	3.40 (0.38)	
University degree	2081	35.3	3.44 (0.39)	
Masters	495	8.4	3.48 (0.40)	
PhD/board certified	178	3.0	3.46 (0.37)	
Fellowship degree	84	1.4	3.52 (0.38)	
Other	1459	24.7	3.39 (0.42)	
Tenure with PHC				< .001
1 year to less than 3 years	1702	27.5	3.44 (0.40)	
3 years to less than 6 years	1822	29.5	3.39 (0.41)	
6 years to less than 11 years	1457	23.6	3.44 (0.37)	
11 years or more	1205	19.5	3.43 (0.39)	
Hours worked per week				< .001
1–4	163	2.7	3.17 (0.35)	
5–16	460	7.6	3.28 (0.42)	
17–24	198	3.3	3.34 (0.45)	
25–32	755	12.4	3.39 (0.43)	
33–40	2046	33.6	3.45 (0.41)	
41 or more	2463	40.5	3.46 (0.35)	
Position				< .001
Primary Care Centre Head	59	1.0	3.56 (0.29)	
Trustee/Administrative Supervisor	121	2.0	3.41 (0.42)	
Receptionist/Medical Records	907	15.0	3.32 (0.42)	
Information Systems Officer/Secretariat	295	4.9	3.35 (0.40)	
Hotel Services Supervisor/Head of Cleaners	57	.9	3.41 (0.28)	
Nursing	1929	31.9	3.48 (0.33)	
General Practitioner	709	11.7	3.50 (0.38)	
Family medicine	191	3.2	3.43 (0.40)	
Physician, other specialities	213	3.5	3.47 (0.43)	
Health Inspector	100	1.7	3.47 (0.35)	
Pharmacist	334	5.5	3.38 (0.41)	
Lab Doctor	274	4.5	3.35 (0.48)	
Technician	625	10.3	3.34 (0.44)	
Phlebotomist	97	1.6	3.32 (0.46)	
Other	136	2.2	3.32 (0.51)	
Department				< .001
Primary Care Clinics	1545	25.7	3.45 (0.37)	
Specialised clinics from the hospitals	99	1.6	3.53 (0.39)	
Preventive health	234	3.9	3.52 (0.33)	
Elderly health clinics	30	0.5	3.36 (0.34)	
School health clinics	68	1.1	3.41 (0.48)	
Dental	602	10.0	3.41 (0.38)	
Nursing	1097	18.3	3.52 (0.29)	
Pharmacy	625	10.4	3.34 (0.44)	
Laboratory	679	11.3	3.32 (0.46)	
Radiology/mammography/dental radiology	57	0.9	3.55 (0.46)	
Administrative	282	4.7	3.36 (0.41)	
Reception and medical records	516	8.6	3.38 (0.43)	
Hotel services	28	0.5	3.35 (0.36)	
Other	142	2.4	3.35 (0.42)	
Attended courses or lectures on patient safety				.052
Yes	3505	60.7	3.43 (0.39)	
No	2269	39.3	3.41 (0.40)	

SD: Standard deviation %: Percentage

*p*: *p*-value (statistically significant if *p* ≤ .05, highly significant if *p* ≤ .001)

### Composite-level results

To determine the areas of strength (positive ratings ≥75%) for the PHCs, and those requiring improvement (positive ratings < 60%), we examined the 10 composites [9, 29]. Only two composites were found to be areas of strength: Teamwork (87.8%) and Organisational Learning (78.8%), whereas five composites were identified to be areas for improvement: Communication about Error (57.7%), Overall Perceptions of Patient Safety and Quality (57.4%), Communication Openness (54.4%), Owner/Managing Partner/Leadership Support for Patient Safety (53.8%), and Work Pressure and Pace (28.4%). The average percentage of positive ratings across all 10 composites is 62.7% (Table 2).

**Table 2:** Percentage of positive ratings for each survey item, composite and outcome compared to international, regional and national benchmarks

Survey item	Kuwait 2018	US 2018		Yemen 2015		Kuwait 2014*	
1. ***Teamwork***	***87.8***	***86.5***	►◄	***96.0***	►◄	***80.3*** ^**a**^	►◄
1.1. When someone in this centre gets really busy, others help out (22)	87.1	86		97		68.0	
1.2. In this centre, there is a good working relationship between staff and providers (23)	88.3	90		97			
1.3. In this centre, we treat each other with respect (26)	92.0	85		96		86.0	
1.4. This centre emphasises teamwork in taking care of patients (34)	83.8	85		94		87.0	
2. ***Work Pressure and Pace***	***28.4***	***46.3***	▼▼	***57.3***	▼▼	***41.0*** ^**b**^	▼▼
2.1. In this centre, we often feel rushed when taking care of patients (24R)	20.1	38		67		24.0	
2.2. We have too many patients for the number of providers in this centre (27R)	12.2	45		58			
2.3. We have enough staff to handle our patient load (32)	50.6	46		49		58.0	
2.4. This centre has too many patients to be able to handle everything effectively (35R)	30.7	56		55			
3. ***Staff Training***	***72.4***	***72.3***	►◄	***68.3***	►◄	**c**	
3.1. This centre trains staff when new processes are put into place (25)	81.2	76		57			
3.2. This centre makes sure staff get the on-the-job training they need (28)	77.8	75		74			
3.3. Staff in this centre are asked to do tasks they haven’t been trained to do (31R)	58.1	66		74			
4. ***Office Processes and Standardisation***	***65.5***	***67.5***	►◄	***64.8***	►◄	**c**	
4.1. This centre is more disorganised than it should be (29R)	59.5	64		46			
4.2. We have good procedures for checking that work in this centre was done correctly (30)	79.0	71		73			
4.3. We have problems with workflow in this centre (33R)	48.3	53		59			
4.4. Staff in this centre follow standardised processes to get tasks done (36)	75.4	82		81			
5. ***Communication Openness***	***54.4***	***69.5***	▼	***58.5***	►◄	***51.0*** ^**a**^	►◄
5.1. Providers in this centre are open to staff ideas about how to improve centre processes (37)	59.2	73		53		70.0	
5.2. Staff are encouraged to express alternative viewpoints in this centre (38)	52.3	73		48		37.0	
5.3. Staff are afraid to ask questions when something does not seem right (40R)	54.1	73		72		46.0	
5.4. It is difficult to voice disagreement in this centre (46R)	51.9	59		61			
6. ***Patient Care Tracking/Follow-up***	***70.6***	***86.3***	▼	***52.3***	▲	**c**	
6.1. This centre reminds patients when they need to schedule an appointment for preventive or routine care (39)	72.9	88		60			
6.2. This centre documents how well our chronic-care patients follow their treatment plans (41)	77.1	80		55			
6.3. Our centre follows up when we do not receive a report we are expecting from an outside provider (42)	51.3	86		26			
6.4. This centre follows up with patients who need monitoring (45)	81.3	91		68			
7. ***Communication about Error***	***57.7***	***72.0***	▼	***67.0***	▼	***51.3*** ^**a**^	▲
7.1. Staff feel like their mistakes are held against them (43R)	33.1	63		67		33.0	
7.2. Providers and staff talk openly about centre problems (44)	57.2	64		79		53.0	
7.3. In this centre, we discuss ways to prevent errors from happening again (47)	72.1	82		74		68.0	
7.4. Staff are willing to report mistakes they observe in this centre (48)	68.3	79		48			
8. ***Owner/Managing Partner/Leadership Support for Patient Safety***	***53.8***	***66.0***	▼	***64.0***	▼	***54.3*** ^**a**^	►◄
8.1. They aren’t investing enough resources to improve the quality of care in this centre (49R)	38.2	47		50		47.0	
8.2. They overlook patient care mistakes that happen over and over (50R)	50.3	78		69		38.0	
8.3. They place a high priority on improving patient care processes (51)	80.7	80		78		78.0	
8.4. They make decisions too often based on what is best for the centre rather than what is best for patients (52R)	45.9	59		59			
9. ***Organisational Learning***	***78.8***	***78.7***	►◄	***83.3***	►◄	***67.0*** ^**b**^	▲
9.1. When there is a problem in our centre, we see if we need to change the way we do things (53)	80.8	83		86			
9.2. This centre is good at changing centre processes to make sure the same problems don’t happen again (57)	78.2	79		64		67.0	
9.3. After this centre makes changes to improve the patient care process, we check to see if the changes worked (59)	77.4	74		100		67.0	
10. ***Overall Perceptions of Patient Safety and Quality***	***57.4***	***77.3***	▼	***76.8***	▼	***30.0*** ^**d**^	▲
10.1. Our centre processes are good at preventing mistakes that could affect patients (54)	76.8	85		87			
10.2. Mistakes happen more than they should in this centre (55R)	65.8	77		98			
10.3. It is just by chance that we don’t make more mistakes that affect our patients (56R)	43.2	77		85			
10.4. In this centre, getting more work done is more important than quality of care (58R)	43.8	70		37		30.0	
***Average patient safety culture percentage across all composites***	***62.7***	***72.1***	▼	***68.4***	►◄	***53.6***	▲
***List of Patient Safety and Quality Issues***	***81.3***	***84.7***	►◄	NR		NA	
A patient was unable to get an appointment within 48 h for an acute/serious problem	79.6	76		NR		NA	
The wrong chart/medical record was used for a patient	84.7	97		NR		NA	
A patient’s chart/medical record was not available when needed	80	93		NR		NA	
Medical information was filed, scanned, or entered into the wrong chart/medical record	86.2	95		NR		NA	
Medical equipment was not working properly or was in need of repair or replacement	76.1	89		NR		NA	
A pharmacy contacted our centre to clarify or correct a prescription	76.2	61		NR		NA	
A patient’s medication list was not updated during his or her visit	80.4	79		NR		NA	
The results from a lab or imaging test were not available when needed	80	79		NR		NA	
A critical abnormal result from a lab or imaging test was not followed up within 1 business day	88.2	93		NR		NA	
***Information Exchange with Other Settings***	***81.4***	***79.8***	►◄	NR		NA	
Outside labs centres?	77.9	79		NR		NA	
Outside imaging centres?	85.3	78		NR		NA	
Pharmacies?	87.4	79		NR		NA	
Hospitals?	82.7	83		NR		NA	
Other?	73.9	NA		NR		NA	
***Overall Ratings on Quality***	***54.5***	***68.8***	***▼***	***56.4***	►◄	NA	
Patient Centred: Is responsive to individual patient preferences, needs, and values	51.7	72		72		NA	
Effective: Is based on scientific knowledge	54.2	72		40		NA	
Timely: Minimises waits and potentially harmful delays	53.2	56		43		NA	
Efficient: Ensures cost-effective care (avoids waste, overuse, and misuse of services)	52.9	61		46		NA	
Equitable: Provides the same quality of care to all individuals regardless of gender, race, ethnicity, socio-economic status, language, etc.	60.6	83		81		NA	
***Overall Rating on Patient Safety:*** Overall, how would you rate the systems and clinical processes your Primary Care Centre has in place to prevent, catch, and correct problems that have the potential to affect patients?	60.4	68	▼	NR		NA	
***Information Exchange within Your Primary Care Centre***	***77.2***	NA		NA		NA	
Primary care centre labs?	78.3	NA		NA		NA	
Imaging services within your Primary Care Centre?	79.2	NA		NA		NA	
Other clinics/physicians?	81.8	NA		NA		NA	
Primary Care Centre pharmacy?	85.2	NA		NA		NA	
Other?	61.3	NA		NA		NA	

The composite-level percentage of responses is the average of composite items percentages

The item-level percentage of responses was calculated using the following formula:

[number of positive responses to the items in the composite/total number of responses to the items in the composite (excluding missing responses)] × 100

The number in parentheses after the item is the question number from the survey R: Negatively worded items that were reverse-coded

▲: Results exceeding the benchmark (greater than + 10%) ►◄: Results meeting the benchmark (between + 10% and − 10%)

▼: Results deviating slightly from the benchmark (between −10% and − 30%) ▼▼: Results deviating greatly from the benchmark (below −30%)

*: Results are selected from comparable items in the HSOPSC conducted at 3 PHCs

a: Three comparable items in the composite b: Two comparable items in the composite c: No comparable items in the composite

d: One comparable items in the composite NA: Not applicable NR: Not reported

### Item-level results

Fifteen items were considered areas of strength, and 19 were identified as areas for improvement. The survey item with the highest positive response rates was “we treat each other with respect” (92.0%). The item with the lowest percentage of positive ratings was “we have too many patients for the number of providers in the centre” (12.2%). An important point to note is that individual items should be examined in the broader context of safety culture, as they are not necessarily significant on their own. All composites, except three—Communication about Error, Communication Openness, and Work Pressure and Pace—had at least one area of strength. All the items comprising the latter two composites were identified as areas requiring improvement.

In a benchmarking exercise, we compared our results to those from the US in 2018 [29], Yemen in 2015 [9] and Kuwait in 2014 [25] (Table 2). The results did not exceed the US benchmark in any composite. However, four composites did meet the US benchmark: Teamwork, Staff Training, Office Processes and Standardisation, and Organisational Learning. The result of the Work Pressure and Pace composite (28.4%) deviated greatly from both the US (46.3%) and Yemen (57.3%) benchmarks. The results exceeded the Yemen benchmark in just one composite: Patient Care Tracking/Follow-up. Our results are either consistent with or exceed previous national findings (Kuwait 2014) with respect to all composites except Work Pressure and Pace. We note that that benchmark was based on a different questionnaire—the HSOPSC—and only similar items were compared. Table 2 also shows a benchmark comparison for other patient safety and quality outcomes.

### Comparison of the PSC composite means of socio-demographic groups

Participants working in Health Region 4, non-specialised clinics, smaller PHCs and rural areas reported the highest PSC percentages, on average. Other groups linked to high mean PSC percentages include those aged over 55 years, Asian nationalities and fellowship degree holders. Table 1 shows a comparison between the socio-demographic groups and the means of “average patient safety culture percentage across all composites”.

### Association between composites and healthcare quality and patient safety outcomes

Using the Kruskal–Wallis test to compare means of composites across the outcomes groups showed that participants who rated aspects of healthcare quality and overall patient safety as “Excellent/Very Good” were associated, with statistical significance, with the highest mean scores for safety culture composites (Table 3). Teamwork received the highest mean score (3.90 ± 0.27) from those giving “Patient Centred”, “Efficient”, and “Overall Rating on Patient Safety” Excellent/Very Good ratings. The Work Pressure and Pace composite scored the lowest (2.65 ± 0.76) among those giving the highest ratings to the “Equitable” area of health care quality.

**Table 3:** Comparison between healthcare quality and patient safety grades and patient safety culture composite scores

	Patient Centred				Effective				Timely				Efficient				Equitable				Overall Rating on Patient Safety			
	a	b	c		a	b	c		a	b	c		a	b	c		a	b	c		a	b	c	
	Mean (SD)	Mean (SD)	Mean (SD)	Sig.	Mean (SD)	Mean (SD)	Mean (SD)	Sig.	Mean (SD)	Mean (SD)	Mean (SD)	Sig.	Mean (SD)	Mean (SD)	Mean (SD)	Sig.	Mean (SD)	Mean (SD)	Mean (SD)	Sig.	Mean (SD)	Mean (SD)	Mean (SD)	Sig.
Teamwork	3.52 (0.56)	3.82 (0.35)	3.90 (0.27)	a-b* a-c* b-c*	3.54 (0.58)	3.80 (0.37)	3.89 (0.26)	a-b* a-c* b-c*	3.62 (0.53)	3.81 (0.36)	3.89 (0.27)	a-b* a-c* b-c*	3.61 (0.53)	3.80 (0.38)	3.90 (0.26)	a-b* a-c* b-c*	3.65 (0.51)	3.81 (0.36)	3.87 (0.30)	a-b* a-c* b-c*	3.46 (0.58)	3.78 (0.39)	3.90 (0.25)	a-b* a-c* b-c*
Work Pressure and Pace	2.45 (0.82)	2.56 (0.73)	2.68 (0.74)	a-b* a-c* b-c*	2.49 (0.80)	2.55 (0.73)	2.66 (0.75)	a-c* b-c*	2.41 (0.77)	2.55 (0.71)	2.70 (0.76)	a-b* a-c* b-c*	2.42 (0.81)	2.56 (0.72)	2.69 (0.74)	a-b* a-c* b-c*	2.47 (0.75)	2.59 (0.72)	2.65 (0.76)	a-b* a-c* b-c	2.36 (0.80)	2.52 (0.74)	2.69 (0.73)	a-b* a-c* b-c*
Staff Training	3.17 (0.71)	2.56 (0.73)	3.74 (0.51)	a-b* a-c* b-c*	3.19 (0.74)	3.52 (0.61)	3.73 (0.52)	a-b* a-c* b-c*	3.30 (0.70)	3.54 (0.59)	3.72 (0.54)	a-b* a-c* b-c*	3.25 (0.73)	3.53 (0.60)	3.74 (0.51)	a-b* a-c* b-c*	3.32 (0.68)	3.53 (0.59)	3.70 (0.56)	a-b* a-c* b-c*	3.09 (0.74)	3.46 (0.63)	3.75 (0.50)	a-b* a-c* b-c*
Office Processes and Standardisation	2.97 (0.71)	3.43 (0.63)	3.70 (0.56)	a-b* a-c* b-c*	3.02 (0.75)	3.39 (0.63)	3.69 (0.57)	a-b* a-c* b-c*	3.12 (0.72)	3.42 (0.62)	3.69 (0.58)	a-b* a-c* b-c*	3.03 (0.73)	3.42 (0.62)	3.70 (0.56)	a-b* a-c* b-c*	3.17 (0.73)	3.45 (0.60)	3.63 (0.61)	a-b* a-c* b-c*	2.78 (0.72)	3.34 (0.62)	3.72 (0.54)	a-b* a-c* b-c*
Communication Openness	2.99 (0.78)	3.34 (0.71)	3.66 (0.70)	a-b* a-c* b-c*	2.96 (0.78)	3.31 (0.73)	3.66 (0.69)	a-b* a-c* b-c*	3.12 (0.80)	3.35 (0.71)	3.63 (0.71)	a-b* a-c* b-c*	3.04 (0.81)	3.35 (0.71)	3.65 (0.69)	a-b* a-c* b-c*	3.06 (0.78)	3.34 (0.69)	3.62 (0.71)	a-b* a-c* b-c*	2.81 (0.77)	3.27 (0.71)	3.67 (0.67)	a-b* a-c* b-c*
Patient Care Tracking/Follow-up	3.18 (0.66)	3.56 (0.54)	3.76 (0.39)	a-b* a-c* b-c*	3.20 (0.69)	3.55 (0.53)	3.74 (0.41)	a-b* a-c* b-c*	3.30 (0.67)	3.56 (0.51)	3.74 (0.43)	a-b* a-c* b-c*	3.24 (0.66)	3.57 (0.52)	3.74 (0.42)	a-b* a-c* b-c*	3.38 (0.63)	3.56 (0.53)	3.70 (0.46)	a-b* a-c* b-c*	3.15 (0.68)	3.50 (0.57)	3.75 (0.41)	a-b* a-c* b-c*
Communication about Error	2.99 (0.65)	3.32 (0.57)	3.52 (0.55)	a-b* a-c* b-c*	2.99 (0.65)	3.30 (0.57)	3.53 (0.54)	a-b* a-c* b-c*	3.08 (0.64)	3.35 (0.57)	3.50 (0.55)	a-b* a-c* b-c*	3.05 (0.65)	3.31 (0.58)	3.53 (0.53)	a-b* a-c* b-c*	3.14 (0.65)	3.35 (0.59)	3.47 (0.56)	a-b* a-c* b-c*	2.83 (0.62)	3.27 (0.58)	3.53 (0.53)	a-b* a-c* b-c*
Leadership Support for Patient Safety	2.94 (0.81)	3.24 (0.67)	3.55 (0.75)	a-b* a-c* b-c*	3.02 (0.82)	3.22 (0.70)	3.54 (0.75)	a-b* a-c* b-c*	3.07 (0.79)	3.22 (0.69)	3.54 (0.75)	a-b* a-c* b-c*	3.01 (0.81)	3.26 (0.68)	3.54 (0.75)	a-b* a-c* b-c*	3.12 (0.76)	3.22 (0.69)	3.50 (0.77)	a-b a-c* b-c*	2.78 (0.78)	3.18 (0.67)	3.56 (0.73)	a-b* a-c* b-c*
Organisational Learning	3.31 (0.66)	3.68 (0.48)	3.83 (0.37)	a-b* a-c* b-c*	3.31 (0.66)	3.67 (0.49)	3.83 (0.37)	a-b* a-c* b-c*	3.44 (0.64)	3.68 (0.48)	3.82 (0.38)	a-b* a-c* b-c*	3.36 (0.64)	3.68 (0.49)	3.83 (0.36)	a-b* a-c* b-c*	3.46 (0.63)	3.68 (0.50)	3.79 (0.41)	a-b* a-c* b-c*	3.17 (0.67)	3.64 (0.49)	3.84 (0.35)	a-b* a-c* b-c*
Overall Perceptions of Patient Safety and Quality	3.04 (0.76)	3.25 (0.67)	3.55 (0.79)	a-b* a-c* b-c*	2.99 (0.75)	3.23 (0.68)	3.57 (0.78)	a-b* a-c* b-c*	3.05 (0.75)	3.25 (0.69)	3.56 (0.77)	a-b* a-c* b-c*	3.06 (0.73)	3.25 (0.70)	3.55 (0.78)	a-b* a-c* b-c*	3.17 (0.76)	3.25 (0.72)	3.48 (0.78)	a-c* b-c*	2.86 (0.77)	3.20 (0.66)	3.56 (0.76)	a-b* a-c* b-c*

Composites scored ranging from 1 to 5.

a: Poor or Fair rating group b: Good rating group c: Excellent or Very Good rating group.

SD: Standard deviation Sig.: Significant difference between groups (*p*-value ≤ .05) * (*p*-value ≤ .001).

Table 4 shows the correlations between all safety culture composites and all healthcare quality and patient safety outcomes, which were all positive except that between the Leadership Support for Patient Safety composite and Information Exchange with Other Settings. Based on Ratner’s interpretation guidelines [31], the correlations are either weak (> 0.00–0.30) or moderate (0.30–0.70). The highest correlation (0.42) is between Office Processes and Standardisation and Overall Rating on Patient Safety. All the healthcare quality and patient safety outcomes have statistically significant correlations with the safety culture composites, except Information Exchange with Other Settings, for which some are not significant.

**Table 4:** Association of patient safety culture composite scores (independent) with patient safety and quality outcomes (dependent)

	Patient Safety and Quality Issues	Information Exchange within Your PHC	Information Exchange with Other Settings	Patient Centred	Effective	Timely	Efficient	Equitable	Overall Rating on Patient Safety
Teamwork	0.21**	0.21**	0.02	0.27**	0.25**	0.23**	0.26**	0.20**	0.32**
Work Pressure and Pace	0.17**	0.08**	0.05*	0.11**	0.09**	0.14**	0.13**	0.08**	0.15**
Staff Training	0.22**	0.21**	0.01	0.28**	0.27**	0.25**	0.27**	0.24**	0.33**
Office Processes and Standardisation	0.28**	0.24**	0.05*	0.34**	0.33**	0.32**	0.35**	0.25**	0.42**
Communication Openness	0.18**	0.19**	0.06**	0.31**	0.32**	0.26**	0.29**	0.28**	0.37**
Patient Care Tracking/Follow-up	0.24**	0.20**	0.05*	0.31**	0.28**	0.28**	0.30**	0.21**	0.31**
Communication about Error	0.18**	0.21**	0.07**	0.28**	0.29**	0.24**	0.28**	0.19**	0.34**
Leadership Support for Patient Safety	0.20**	0.15**	−0.01	0.28**	0.25**	0.24**	0.24**	0.20**	0.33**
Organisational Learning	0.24**	0.18**	0.02	0.32**	0.31**	0.27**	0.31**	0.22**	0.38**
Overall Perceptions of Patient Safety and Quality	0.21**	0.18**	0.02	0.25**	0.28**	0.27**	0.25**	0.17**	0.32**
***Average across all composites***	0.33**	0.29**	0.06**	0.42**	0.41**	0.39**	0.42**	0.32**	0.50**

* Correlation is significant at the .05 level

** Correlation is highly significant at the .001 level

### Multiple regression analysis

Tables 5 and 6 show the results of the regression modelling. Given that the *R*-squared value, expressed as a percentage, represents the variation in the outcome that can be explained by the model, the socio-demographics (Table 5) account for 1–9% of the variability in the 10 safety culture composites. The predictors listed in Table 6 have greater capacity to predict outcomes, accounting for 14–37% of the variability in the healthcare quality and patient safety outcomes. It is worth reiterating that the two tables report only the predictor variables that were included in each regression model. The two tables report the constant value used to calculate the score of each outcome. They also report the regression coefficients of the predictors, which represents by how much the outcome variable will increase or decrease as a result of a unit change in the predictor variable.

**Table 5:** Predictors of patient safety culture composite scores

*R*^2^	Teamwork		Work Pressure and Pace		Staff Training		Office Processes & Standardisation		1Communication Openness		Patient Care Tracking/Follow-up		Communication about Error		Leadership Support for Patient Safety		Organisational Learning		Overall Perceptions of Patient Safety and Quality	
	0.03		0.04		0.03		0.06		0.03		0.02		0.09		0.01		0.03		0.04	
	Beta	(SE)	Beta	(SE)	Beta	(SE)	Beta	(SE)	Beta	(SE)	Beta	(SE)	Beta	(SE)	Beta	(SE)	Beta	(SE)	Beta	(SE)
Constant	3.90	(0.01)	3.33	(0.15)	3.59	(0.03)	3.58	(0.03)	3.47	(0.03)	3.52	(0.03)	3.65	(0.01)	3.34	(0.01)	3.66	(0.04)	3.45	(0.03)
Location																				
Urban			−0.82**	(0.15)			−0.07*	(0.02)												
Suburban			−0.59**	(0.15)					0.06*	(0.03)	0.07**	(0.02)					0.04**	(0.02)		
Rural			0				0		0		0						0			
Language																				
Arabic	−0.09**	(0.01)			−0.07*	(0.03)	−0.16**	(0.03)	−0.15**	(0.02)	−0.01	(0.03)	−0.38**	(0.02)			−0.05	(0.03)	−0.09*	(0.03)
English	0				0		0		0		0		0				0		0	
Work experience																				
1 to < 3 yrs	0		0		0		0										0		0	
3 to < 6 yrs	− 0.03*	(0.01)	−0.11**	(0.03)			−0.06*	(0.02)											−0.06*	(0.03)
6 to < 11 yrs			−0.15**	(0.03)													0.06*	(0.02)		
≥ 11 yrs			−0.18**	(0.03)	0.05*	(0.02)											0.07**	(0.02)		
Working hours																				
1–4 h/w	−0.20**	(0.03)	0.14*	(0.07)	−0.28**	(0.06)	− 0.19*	(0.06)	− 0.38**	(0.07)	−0.21**	(0.05)	−0.18**	(0.05)			−0.24**	(0.05)	−0.51**	(0.07)
5–16 h/w	− 0.09**	(0.02)			−0.17**	(0.03)	−0.09*	(0.04)	−0.16**	(0.04)			−0.12**	(0.03)	−0.19**	(0.05)	−0.07*	(0.03)	−0.31**	(0.05)
17–24 h/w	− 0.14**	(0.03)									−0.13*	(0.04)								
25–32 h/w			0.05	(0.03)	−0.06*	(0.03)			0.09*	(0.04)										
33–40 h/w			0.08**	(0.02)			0.05*	(0.02)	0.09**	(0.02)									0.09**	(0.03)
41 h/w	0		0		0		0		0		0		0		0		0		0	
Nationality																				
Kuwaiti	0		0		0		0		0		0				0		0		0	
Arabian	0.03*	(0.01)	−0.09**	(0.02)	0.12**	(0.02)			0.10**	(0.03)	0.05*	(0.02)			0.14**	(0.03)	0.06*	(0.02)	0.13**	(0.03)
Asian					0.09*	(0.03)	0.19**	(0.04)			0.12**	(0.03)					0.09*	(0.03)		

*R*^2^: *R*-squared value Beta: Regression coefficient SE: Standard error yrs.: years.

hrs/w: hours per week * Regression coefficient is significant (*p*-value ≤ .05) ** Regression coefficient is highly significant (*p*-value ≤ .001).

The composite-level percentage of responses is the average of composite item percentages.

**Table 6:** Predictors of patient safety and quality outcomes (dependent variables)

*R*^2^	Patient Safety and Quality Issue†		Information Exchange within Your PHC†		Patient Centred‡		Effective‡		Timely‡		Efficient‡		Equitable‡		Overall Rating on Patient Safety‡	
	0.19		0.14		0.26		0.26		0.21		0.24		0.18		0.37	
	Beta	(SE)	Beta	(SE)	Beta	(SE)	Beta	(SE)	Beta	(SE)	Beta	(SE)	Beta	(SE)	Beta	(SE)
Constant	−9.02	(5.39)	14.71	(5.77)	−1.00	(0.15)	−0.83	(0.16)	−1.25	(0.23)	− 1.44	(0.22)	−0.94	(0.25)	− 1.82	(0.18)
Patient safety culture composites																
Teamwork	4.30*	(1.47)	6.54**	(1.52)					0.14*	(0.06)	0.13*	(0.06)	0.17*	(0.07)	0.14*	(0.05)
Work Pressure and Pace	2.23**	(0.61)							0.15**	(0.03)	0.06*	(0.03)	0.06*	(0.03)	0.05*	(0.02)
Staff Training	2.93**	(0.88)			0.10*	(0.03)	0.16**	(0.03)	0.10*	(0.04)	0.12*	(0.04)	0.20**	(0.04)	0.10*	(0.03)
Office Processes and Standardisation	3.42**	(0.90)	2.38*	(0.85)			0.16**	(0.03)	0.14**	(0.04)	0.14**	(0.04)	0.17**	(0.05)	0.26**	(0.03)
Communication Openness					0.16**	(0.03)	0.17**	(0.03)	0.08*	(0.03)	0.15**	(0.03)	0.29**	(0.04)	0.17**	(0.03)
Patient Care Tracking/Follow-up	5.70**	(0.95)	5.07**	(0.99)	0.42**	(0.04)	0.31**	(0.04)	0.35**	(0.04)	0.36**	(0.04)	0.24**	(0.05)	0.26**	(0.03)
Communication about Error			1.46	0.93												
Leadership Support for Patient Safety					0.10**	(0.02)			0.08*	(0.03)	0.09*	(0.03)	0.10*	(0.03)	0.16**	(0.02)
Organisational Learning	6.64**	(1.08)	4.55**	(1.15)	0.32**	(0.04)	0.27**	(0.04)	0.16**	(0.05)	0.28**	(0.05)			0.33**	(0.04)
Overall Perceptions of Patient Safety and Quality	2.00*	(0.64)	1.70*	(0.66)			0.12**	(0.03)	0.12**	(0.03)	0.06*	(0.03)			0.06*	(0.02)
Language																
Arabic	−5.78**	(1.03)	−6.32**	(1.08)			0.08*	(0.04)	0.15**	(0.05)			0.38**	(0.05)	0.08*	(0.04)
English	0		0				0		0				0		0	
Working hours																
1–4 h/w	−6.22*	(3.01)							−0.24*	(0.12)						
5–16 h/w			−4.29*	(1.69)									0.18*	(0.08)		
17–24 h/w	−6.64*	(2.34)											0.26*	(0.12)		
25–32 h/w									−0.17*	(0.06)						
33–40 h/w																
41 h/w	0		0						0				0			

†: Item scored as percentage ‡: Item scored from 1 to 5

*R*^2^: *R*-squared value Beta: Regression coefficient SE: Standard error

* Regression coefficient is significant (*p*-value ≤ .05) ** Regression coefficient is highly significant (*p*-value ≤ .001) hrs/w: hours per week

The composite-level percentage of responses is the average of composite item percentages

Of all the regressed socio-demographics, only working hours can predict the score of the 10 safety composites (Table 5). There are 24 instances in which the socio-demographic variables increased the safety culture composite scores, whereas scores decreased in 37 cases.

Table 6 shows that only language and working hours can predict healthcare quality and patient safety outcomes. However, Patient Centred and Efficient grades are not predicted by these two regressed socio-demographics. In addition, working hours do not predict the Effective grade. The Patient Care Tracking/Follow-up composite predicts all the healthcare quality and patient safety outcomes, whereas the Communication about Error composite predicts the ratings of only one healthcare quality and patient safety outcome: Information Exchange within Your Primary Care Centre. Unlike the two regressed socio-demographics, a one-unit increase in the score of any safety culture composite resulted in higher grades and percentages of all healthcare quality and patient safety outcomes.

## Discussion

The literature reports the use of different approaches to assessing the culture of safety, although questionnaire-based quantitative approaches are the most frequently used [1, 8, 32–37]. For hospital settings, the highest cited tool is the HSOPSC [2, 24, 30, 38–40]; this is also the case for primary care settings [1, 13, 25, 41]. Recently, the use of another version of this tool, the MOSPSC, has emerged [9, 28, 42–44].

We decided to use the MOSPSC in this study as the PHC setting provides primary care services comparable to the ambulatory services offered in medical offices in the US. In contrast to Kuwait, where 85.1% of PHCs have more than 50 members of staff, 93% of medical offices in the US—the country in which the MOSPSC was devised—have fewer than 20. Furthermore, all Kuwait PHCs have more than one speciality, whereas 75% of US medical offices operate under a single speciality. These differences urge us to reflect on which is a more suitable tool for the PHC setting in Kuwait: the hospital or medical office version? A detailed factor analysis might give a clear answer, but this was outside the scope of the present study. Moreover, these differences in the scale and speciality of care settings between the two countries add complexity and multiply the communication channels in the Kuwaiti settings relative to those the MOSPSC was designed to survey. This was reflected in the modifications we introduced to the tool by adding a five-item section to assess information exchange within a PHC. It also impacted the participant responses, where the rate of response to this newly added section (36.2–57.5%) was significantly higher than the responses to the original MOSPSC section “Information Exchange with Other Settings” (29.4–39.9%).

This study revealed five areas for improvement. Two of these—Communication about Error and Communication Openness—could be improved by institutionalising effective communication practices. Owner/Managing Partner/Leadership Support for Patient Safety could be improved with more effective governance and leadership. The literature consensus is that these three areas do require improvement. Poor communication is linked to unsafe worker behaviours, which include violations of policies and procedures and poor reporting of events [45]. Establishing an open communication system in PHCs is an important factor in enhancing patient safety outcomes [36]. Good communication motivates healthcare workers to learn effectively from their mistakes and adjust their practices accordingly [46]. Front-line staff should feel that communications with managers are heard and acknowledged [47]. Leadership is an essential component in propagating a free and supportive environment for the reporting of safety issues [48]. Providing feedback or closing the loop builds trust and openness, which are important properties of a healthy PSC [47].

Improvement of a fourth area—Work Pressure and Pace—is vital for two reasons. First, it had the lowest score of the 10 composites and was far below (below −30%) the international, regional and national benchmarks. Second, such a low score seemed anomalous in the Kuwaiti context. In a high-income country such as Kuwait, the public healthcare system should have the resources available for matching its capacity to public demand and for attracting competent professionals into the country, even amidst a highly competitive regional healthcare market. Also, 59.5% of study participants are working 40 h per week or less, compared to only 14% in the US [49]. One cannot confidently conclude that the negative feelings towards Work Pressure and Pace in Kuwait reflects a genuine high-workload situation created by the fewer working hours, or reflects other reasons. However, adverse work conditions can create several limitations in a healthcare setting such as creating a chaotic work environment, improper communication with patients or staff, dissatisfaction, burnout, and a lower standard of care may result [50, 51]. In general, this issue can be addressed by enhancing team functioning through assigning tasks to lessen the pressure on physicians, including having their administrative work performed by medical assistants so they can increase their face-to-face time with patients [52]. Additional interventions that require further evaluation but might be useful in reducing burnout are: creating standing order sets, providing responsive information technology support, offering flexible or part-time working schedules, hiring locum clinicians to cover unexpected leave, and building workplace teams that address workflow and quality challenges.

The fifth area for improvement is Overall Perceptions of Patient Safety and Quality. Such perceptions are critical factors in enhancing organisational aspects and quality outcomes in PHCs [13, 36, 46]. In primary care, staff perception is affected by the belief that PHCs have “low-risk” potential to cause neglect to safety and quality, which might lead to the development of unexpected dangers [36]. These challenges may be addressed by regularly assessing the culture surrounding safety and quality to test the effectiveness of safety interventions [1], engaging patients and families in their care plan to achieve high-quality, safe and effective care [53], and tackling problematic attitudes and practices such as punitive responses to errors and miscommunication, and ensuring that patient safety goals and objectives are part of strategic and operational plans [1].

Another unexpected finding is the non-significant relationship between attending training on safety and the PSC percentage. This contradiction with established evidence [48] brings these training programmes into question with respect to needs assessment, objectives and content, and impact evaluation. Respondents to the English version of the tool showed a significantly higher mean percentage. This finding can be attributed to these courses being delivered in English, which likely facilitates a better understanding of the related questionnaire items. However, such claims should be subject to much caution considering the non-significant relationship between safety training attendance and the PSC percentage.

Although there are significant differences between groups in the majority of socio-demographics, only some of these variables can predict the grades of a composite, and fewer still can predict patient safety and quality outcome ratings. Furthermore, even for those few predictors, multiple regression modelling demonstrates a limited contribution to the outcome variable scores.

### Strengths and limitations

This study has several strengths. It is the first nationwide study in Kuwait or the region to assess PSC in public PHCs. It involved a relatively large sample size representing different professions and authority levels. The cross-sectional design allowed different variables in the population sample to be measured at a single point of time for gathering accurate data that are less prone to the potential bias of case series and case reports [54]. Also, the study used an internationally recognised validated tool, which allowed international benchmarking.

However, some limitations exist. The study is designed to determine relationships between variables, not to imply causality between them. Because of the limited use of the MOSPSC in regional primary care settings, the only available study is from Yemen [9], which is not the most comparable context to Kuwait. Furthermore, using paper-based questionnaires is not environmentally friendly and required considerable effort in data entry and cleaning. In addition, it allowed participants to skip questions, resulting in many missing responses.

### Practice and research implications

The overall objective of this study is to help PHCs promote patient safety practices by establishing a healthy PSC and strengthening their systems to prevent adverse events and mitigate their impact. Other beneficiaries from this study are patients, who stand to gain indirectly from strategies that improve the PSC. Furthermore, the results and lessons learned can help other Eastern Mediterranean countries wishing to have comparable healthcare systems formulate strategies and promote a PSC that is unique to their context. Investment in robust training programmes on patient safety should be informed by these study findings if tangible improvements are to be made.

This study signals a need for qualitative and mixed-methods research to better understand the topic of patient safety, especially with the limited predictability of the outcome variables in this quantitative approach. We recommend the use of electronic data collection in future studies, especially for large samples. Further research should investigate perceptions about workload and work pressures. Also, researchers are encouraged to assess the suitability of the MOSPSC for primary care settings of comparable size and level of speciality.

## Conclusions

Assessing and improving the culture around patient safety is paramount if a PHC aims to improve the quality and safety of the clinical services it provides. The culture within a PHC is reflected in the decisions made concerning safety, and this has an impact on patient outcomes. Our evaluation of these results against international and regional benchmarks is expected to help healthcare leaders in Kuwait to better visualise performance and set realistic targets for improvement. As such, the findings of this study should enlighten and lead national strategies aimed at improving patient safety governance and practices.

## Supplementary Information

**Additional file 1.**

**Additional file 2.**

## Data Availability

The datasets generated and/or analysed during the current study are not publicly available owing to MOH restrictions. However, datasets are available from the corresponding author upon reasonable request.

## Abbreviations

AHRQ: Agency for Healthcare Research and Quality
HSOPSC: Hospital Survey on Patient Safety Culture
MOH: Ministry of Health
MOSPSC: Medical Office Survey on Patient Safety Culture
PHC: Primary Healthcare Centre
PSC: patient safety culture
SD: standard deviation
SE: standard error
US: United States
