# Correction to: Baseline assessment of patient safety culture in primary care centres in Kuwait: a national cross-sectional study

**DOI:** 10.1186/s12913-021-07280-9

**Published:** 2021-11-24

**Authors:** Talal ALFadhalah, Buthaina Al Mudaf, Hanaa A. Alghanim, Gheed Al Salem, Dina Ali, Hythem M. Abdelwahab, Hossam Elamir

**Affiliations:** 1 Quality and Accreditation Directorate, Ministry of Health, Kuwait City, Kuwait; 2 Assistant Undersecretary of Public Health Affairs, Ministry of Health, Kuwait City, Kuwait; 3 Safety Department, Quality and Accreditation Directorate, Ministry of Health, Kuwait City, Kuwait; 4 Accreditation Affairs Department, Quality and Accreditation Directorate, Ministry of Health, Kuwait City, Kuwait; 5 National Blood Transfusion Services, Ministry of Health and Population, Giza, Egypt; 6 Research and Technical Support Department, Quality and Accreditation Directorate, Ministry of Health, Kuwait City, Kuwait

**Correction to: BMC Health Serv Res 21, 1172 (2021)**

**https://doi.org/10.1186/s12913-021-07199-1**

Following publication of the original article [1], it was noted that due to a typesetting error the signs”►◄” were removed in Table 2 and its footnote of the pdf version.

**Table 2:** Percentage of positive ratings for each survey item, composite and outcome compared to international, regional and national benchmarks

Survey item	Kuwait 2018	US 2018		Yemen 2015		Kuwait 2014*	
***1. Teamwork***	***87.8***	***86.5***	►◄	***96.0***	►◄	***80.3*** ^a^	►◄
1.1. When someone in this centre gets really busy, others help out (22)	87.1	86		97		68.0	
1.2. In this centre, there is a good working relationship between staff and providers (23)	88.3	90		97			
1.3. In this centre, we treat each other with respect (26)	92.0	85		96		86.0	
1.4. This centre emphasises teamwork in taking care of patients (34)	83.8	85		94		87.0	
***2. Work Pressure and Pace***	***28.4***	***46.3***	▼▼	***57.3***	▼▼	***41.0*** ^b^	▼▼
2.1. In this centre, we often feel rushed when taking care of patients (24R)	20.1	38		67		24.0	
2.2. We have too many patients for the number of providers in this centre (27R)	12.2	45		58			
2.3. We have enough staff to handle our patient load (32)	50.6	46		49		58.0	
2.4. This centre has too many patients to be able to handle everything effectively (35R)	30.7	56		55			
***3. Staff Training***	***72.4***	***72.3***	►◄	***68.3***	►◄	^c^	
3.1. This centre trains staff when new processes are put into place (25)	81.2	76		57			
3.2. This centre makes sure staff get the on-the-job training they need (28)	77.8	75		74			
3.3. Staff in this centre are asked to do tasks they haven’t been trained to do (31R)	58.1	66		74			
***4. Office Processes and Standardisation***	***65.5***	***67.5***	►◄	***64.8***	►◄	^c^	
4.1. This centre is more disorganised than it should be (29R)	59.5	64		46			
4.2. We have good procedures for checking that work in this centre was done correctly (30)	79.0	71		73			
4.3. We have problems with workflow in this centre (33R)	48.3	53		59			
4.4. Staff in this centre follow standardised processes to get tasks done (36)	75.4	82		81			
***5. Communication Openness***	***54.4***	***69.5***	▼	***58.5***	►◄	***51.0*** ^a^	►◄
5.1. Providers in this centre are open to staff ideas about how to improve centre processes (37)	59.2	73		53		70.0	
5.2. Staff are encouraged to express alternative viewpoints in this centre (38)	52.3	73		48		37.0	
5.3. Staff are afraid to ask questions when something does not seem right (40R)	54.1	73		72		46.0	
5.4. It is difficult to voice disagreement in this centre (46R)	51.9	59		61			
***6. Patient Care Tracking/Follow-up***	***70.6***	***86.3***	▼	***52.3***	▲	^c^	
6.1. This centre reminds patients when they need to schedule an appointment for preventive or routine care (39)	72.9	88		60			
6.2. This centre documents how well our chronic-care patients follow their treatment plans (41)	77.1	80		55			
6.3. Our centre follows up when we do not receive a report we are expecting from an outside provider (42)	51.3	86		26			
6.4. This centre follows up with patients who need monitoring (45)	81.3	91		68			
***7. Communication about Error***	***57.7***	***72.0***	▼	***67.0***	▼	***51.3*** ^a^	▲
7.1. Staff feel like their mistakes are held against them (43R)	33.1	63		67		33.0	
7.2. Providers and staff talk openly about centre problems (44)	57.2	64		79		53.0	
7.3. In this centre, we discuss ways to prevent errors from happening again (47)	72.1	82		74		68.0	
7.4. Staff are willing to report mistakes they observe in this centre (48)	68.3	79		48			
***8. Owner/Managing Partner/Leadership Support for Patient Safety***	***53.8***	***66.0***	▼	***64.0***	▼	***54.3*** ^a^	►◄
8.1. They aren’t investing enough resources to improve the quality of care in this centre (49R)	38.2	47		50		47.0	
8.2. They overlook patient care mistakes that happen over and over (50R)	50.3	78		69		38.0	
8.4. They place a high priority on improving patient care processes (51)	80.7	80		78		78.0	
8.5. They make decisions too often based on what is best for the centre rather than what is best for patients (52R)	45.9	59		59			
***9. Organisational Learning***	***78.8***	***78.7***	►◄	***83.3***	►◄	***67.0*** ^b^	▲
9.1. When there is a problem in our centre, we see if we need to change the way we do things (53)	80.8	83		86			
9.2. This centre is good at changing centre processes to make sure the same problems don’t happen again (57)	78.2	79		64		67.0	
9.3. After this centre makes changes to improve the patient care process, we check to see if the changes worked (59)	77.4	74		100		67.0	
***10. Overall Perceptions of Patient Safety and Quality***	***57.4***	***77.3***	▼	***76.8***	▼	***30.0*** ^d^	▲
10.1. Our centre processes are good at preventing mistakes that could affect patients (54)	76.8	85		87			
10.2. Mistakes happen more than they should in this centre (55R)	65.8	77		98			
10.3. It is just by chance that we don’t make more mistakes that affect our patients (56R)	43.2	77		85			
10.4. In this centre, getting more work done is more important than quality of care (58R)	43.8	70		37		30.0	
***Average patient safety culture percentage across all composites***	***62.7***	***72.1***	▼	***68.4***	►◄	***53.6***	▲
***List of Patient Safety and Quality Issues***	***81.3***	***84.7***	►◄	NR		NA	
A patient was unable to get an appointment within 48 h for an acute/serious problem	79.6	76		NR		NA	
The wrong chart/medical record was used for a patient	84.7	97		NR		NA	
A patient’s chart/medical record was not available when needed	80	93		NR		NA	
Medical information was filed, scanned, or entered into the wrong chart/medical record	86.2	95		NR		NA	
Medical equipment was not working properly or was in need of repair or replacement	76.1	89		NR		NA	
A pharmacy contacted our centre to clarify or correct a prescription	76.2	61		NR		NA	
A patient’s medication list was not updated during his or her visit	80.4	79		NR		NA	
The results from a lab or imaging test were not available when needed	80	79		NR		NA	
A critical abnormal result from a lab or imaging test was not followed up within 1 business day	88.2	93		NR		NA	
***Information Exchange with Other Settings***	***81.4***	***79.8***	►◄	NR		NA	
Outside labs centres?	77.9	79		NR		NA	
Outside imaging centres?	85.3	78		NR		NA	
Pharmacies?	87.4	79		NR		NA	
Hospitals?	82.7	83		NR		NA	
Other?	73.9	NA		NR		NA	
***Overall Ratings on Quality***	***54.5***	***68.8***	***▼***	***56.4***	►◄	NA	
Patient Centred: Is responsive to individual patient preferences, needs, and values	51.7	72		72		NA	
Effective: Is based on scientific knowledge	54.2	72		40		NA	
Timely: Minimises waits and potentially harmful delays	53.2	56		43		NA	
Efficient: Ensures cost-effective care (avoids waste, overuse, and misuse of services)	52.9	61		46		NA	
Equitable: Provides the same quality of care to all individuals regardless of gender, race, ethnicity, socio-economic status, language, etc.	60.6	83		81		NA	
***Overall Rating on Patient Safety:*** Overall, how would you rate the systems and clinical processes your Primary Care Centre has in place to prevent, catch, and correct problems that have the potential to affect patients?	60.4	68	▼	NR		NA	
***Information Exchange within Your Primary Care Centre***	***77.2***	NA		NA		NA	
Primary care centre labs?	78.3	NA		NA		NA	
Imaging services within your Primary Care Centre?	79.2	NA		NA		NA	
Other clinics/physicians?	81.8	NA		NA		NA	
Primary Care Centre pharmacy?	85.2	NA		NA		NA	
Other?	61.3	NA		NA		NA	

The composite-level percentage of responses is the average of composite items percentages.

The item-level percentage of responses was calculated using the following formula: [number of positive responses to the items in the composite/total number of responses to the items in the composite (excluding missing responses)] × 100.

The number in parentheses after the item is the question number from the survey.

R: Negatively worded items that were reverse-coded.

▲: Results exceeding the benchmark (greater than + 10%).

►◄: Results meeting the benchmark (between + 10% and − 10%).

▼: Results deviating slightly from the benchmark (between −10% and − 30%).

▼▼: Results deviating greatly from the benchmark (below −30%).

*: Results are selected from comparable items in the HSOPSC conducted at 3 PHCs

^a^Three comparable items in the composite

^b^Two comparable items in the composite

^c^No comparable items in the composite

^d^One comparable items in the composite

*NA*: Not applicable.

*NR*: Not reported.

The correct table has been included in this correction, and the original article has been corrected.
