# Supplementary material for: Baseline assessment of patient safety culture in primary care centres in Kuwait: a national cross-sectional study
Source: BMC Health Serv Res. 2021 Oct 28;21:1172. doi: 10.1186/s12913-021-07199-1 (PMC8555195; doi:10.1186/s12913-021-07199-1)
Supplement: Supplementary file 1 — Additional file 1. [file 12913_2021_7199_MOESM1_ESM.pdf]

Code:

Primary Care Center
Survey on Patient Safety

SURVEY INSTRUCTIONS

Think about the way things are done in your Primary Care Center and provide your opinions on issues that affect the overall safety and quality of the care provided to patients in your center.

In this survey, the term **provider** refers to physicians and nurses who diagnose, treat patients, and prescribe medications. The term **staff** refers to all others who work in the center.

- If a question does not apply to you or you don't know the answer, please check "Does Not Apply or Don't Know."
- If you work in more than one center, when answering this survey answer only about the center where you received this survey—do not answer about your entire practice.
- If your center is in a building with other Primary Care Centers, answer only about the center you belong to.

SECTION A: **Patient Safety and Quality Issues**

The following section describe things that can happen in Primary Care Centers that affect patient safety and quality of care. **In your opinion, how often did the following things happen in your center *OVER THE PAST 12 MONTHS*?**

	Daily	Weekly	Monthly	Several times in the past 12 months	Once or twice in the past 12 months	Not in the past 12 months	Does Not Apply or Don't Know
**Access to Care**							
1. A patient was unable to get an appointment within 48 hours for an acute/serious problem	☐1	☐2	☐3	☐4	☐5	☐6	☐9
**Patient Identification**							
2. The wrong chart/medical record was used for a patient	☐1	☐2	☐3	☐4	☐5	☐6	☐9
**Charts/Medical Records**							
3. A patient's chart/medical record was not available when needed	☐1	☐2	☐3	☐4	☐5	☐6	☐9
4. Medical information was filed, scanned, or entered into the wrong chart/medical record	☐1	☐2	☐3	☐4	☐5	☐6	☐9
**Medical Equipment**							
5. Medical equipment was not working properly or was in need of repair or replacement	☐1	☐2	☐3	☐4	☐5	☐6	☐9

SECTION A: **List of Patient Safety and Quality Issues** (continued)

How often did the following things happen in your Primary Care Center *<u>OVER THE PAsST 12 MONTHS</u>*?

	Daily	Weekly	Monthly	Several times in the past 12 months	Once or twice in the past 12 months	Not in the past 12 months	Does Not Apply or Don't Know
**Medication**							
6. A pharmacy contacted our Center to clarify or correct a prescription	☐1	☐2	☐3	☐4	☐5	☐6	☐9
7. A patient's medication list was not updated during his or her visit	☐1	☐2	☐3	☐4	☐5	☐6	☐9
**Diagnostics & Tests**							
8. The results from a lab or imaging test were not available when needed	☐1	☐2	☐3	☐4	☐5	☐6	☐9
9. A critical abnormal result from a lab or imaging test was not followed up within 1 business day	☐1	☐2	☐3	☐4	☐5	☐6	☐9

SECTION B: **Information Exchange Within your Primary Care Center**

Over the past 12 months, how often has your Primary Care Center had problems exchanging accurate, complete, and timely information with:

	Problems daily	Problems weekly	Problems monthly	Problems several times in the past 12 months	Problems once or twice in the past 12 months	No problems in the past 12 months	Does Not Apply or Don't Know
10. Primary care center labs?	☐1	☐2	☐3	☐4	☐5	☐6	☐9
11. Imaging services within your Primary care center?	☐1	☐2	☐3	☐4	☐5	☐6	☐9
12. Other clinics/ physicians?	☐1	☐2	☐3	☐4	☐5	☐6	☐9
13. Primary care center pharmacy?	☐1	☐2	☐3	☐4	☐5	☐6	☐9
Other ? (Specify): 14. ……………………	☐1	☐2	☐3	☐4	☐5	☐6	☐9
15. ……………………	☐1	☐2	☐3	☐4	☐5	☐6	☐9

SECTION C: **Information Exchange With Other Settings**

Over the past 12 months, how often has your Primary Care Center had problems exchanging accurate, complete, and timely information with:

	Problems daily	Problems weekly	Problems monthly	Problems several times in the past 12 months	Problems once or twice in the past 12 months	No problems in the past 12 months	Does Not Apply or Don't Know
16. Outside labs centers?	☐1	☐2	☐3	☐4	☐5	☐6	☐9
17. Outside imaging centers?	☐1	☐2	☐3	☐4	☐5	☐6	☐9
18. Pharmacies?	☐1	☐2	☐3	☐4	☐5	☐6	☐9
19. Hospitals?	☐1	☐2	☐3	☐4	☐5	☐6	☐9
Other? (Specify): 20.	☐1	☐2	☐3	☐4	☐5	☐6	☐9
Other? (Specify): 21.	☐1	☐2	☐3	☐4	☐5	☐6	☐9

SECTION D: **Working in Your Primary Care Center**

**How much do you agree or disagree with the following statements?**	Strongly Disagree	Disagree	Neither Agree nor Disagree	Agree	Strongly Agree	Does Not Apply or Don't Know
22. When someone in this Center gets really busy, others help out	☐1	☐2	☐3	☐4	☐5	☐9
23. In this Center, there is a good working relationship between staff and providers	☐1	☐2	☐3	☐4	☐5	☐9
24. In this Center, we often feel rushed when taking care of patients	☐1	☐2	☐3	☐4	☐5	☐9
25. This Center trains staff when new processes are put into place	☐1	☐2	☐3	☐4	☐5	☐9
26. In this Center, we treat each other with respect	☐1	☐2	☐3	☐4	☐5	☐9
27. We have too many patients for the number of providers in this Center	☐1	☐2	☐3	☐4	☐5	☐9
28. This Center makes sure staff get the on-the-job training they need	☐1	☐2	☐3	☐4	☐5	☐9
29. This Center is more disorganized than it should be	☐1	☐2	☐3	☐4	☐5	☐9

How much do you agree or disagree with the following statements?	Strongly Disagree	Disagree	Neither Agree nor Disagree	Agree	Strongly Agree	Does Not Apply or Don't Know
30. We have good procedures for checking that work in this Center was done correctly	☐1	☐2	☐3	☐4	☐5	☐9
31. Staff in this Center are asked to do tasks they haven't been trained to do	☐1	☐2	☐3	☐4	☐5	☐9
32. We have enough staff to handle our patient load	☐1	☐2	☐3	☐4	☐5	☐9
33. We have problems with workflow in this Center	☐1	☐2	☐3	☐4	☐5	☐9
34. This Center emphasizes teamwork in taking care of patients	☐1	☐2	☐3	☐4	☐5	☐9
35. This Center has too many patients to be able to handle everything effectively	☐1	☐2	☐3	☐4	☐5	☐9
36. Staff in this Center follow standardized processes to get tasks done	☐1	☐2	☐3	☐4	☐5	☐9

SECTION E: **Communication and Follow-up**

How often do the following things happen in your Primary Care Center?	Never	Rarely	Some-times	Most of the time	Always	Does Not Apply or Don't Know
37. Providers in this Center are open to staff ideas about how to improve Center processes	☐1	☐2	☐3	☐4	☐5	☐9
38. Staff are encouraged to express alternative viewpoints in this Center	☐1	☐2	☐3	☐4	☐5	☐9
39. This Center reminds patients when they need to schedule an appointment for preventive or routine care	☐1	☐2	☐3	☐4	☐5	☐9
40. Staff are afraid to ask questions when something does not seem right	☐1	☐2	☐3	☐4	☐5	☐9
41. This Center documents how well our chronic-care patients follow their treatment plans	☐1	☐2	☐3	☐4	☐5	☐9
42. Our Center follows up when we do not receive a report we are expecting from an outside provider	☐1	☐2	☐3	☐4	☐5	☐9
43. Staff feel like their mistakes are held against them	☐1	☐2	☐3	☐4	☐5	☐9
44. Providers and staff talk openly about Center problems	☐1	☐2	☐3	☐4	☐5	☐9

How often do the following things happen in your Primary Care Center?	Never	Rarely	Some-times	Most of the time	Always	Does Not Apply or Don't Know
45. This Center follows up with patients who need monitoring	☐1	☐2	☐3	☐4	☐5	☐9
46. It is difficult to voice disagreement in this Center	☐1	☐2	☐3	☐4	☐5	☐9
47. In this Center, we discuss ways to prevent errors from happening again	☐1	☐2	☐3	☐4	☐5	☐9
48. Staff are willing to report mistakes they observe in this Center	☐1	☐2	☐3	☐4	☐5	☐9

SECTION F: **Owner/Managing Partner/Leadership Support**

A. Are you an owner, a managing partner, or in a leadership position with responsibility for making financial decisions for your Primary Care Center?

☐1 Yes → ***Go to Section G***

☐2 No → ***Continue below***

How much do you agree or disagree with the following statements about the <u>owners/ managing partners/leadership of your Primary Care Center?</u>	Strongly Disagree	Disagree	Neither Agree nor Disagree	Agree	Strongly Agree	Does Not Apply or Don't Know
49. They aren't investing enough resources to improve the quality of care in this Center	☐1	☐2	☐3	☐4	☐5	☐9
50. They overlook patient care mistakes that happen over and over	☐1	☐2	☐3	☐4	☐5	☐9
51. They place a high priority on improving patient care processes.	☐1	☐2	☐3	☐4	☐5	☐9
52. They make decisions too often based on what is best for the Center rather than what is best for patients	☐1	☐2	☐3	☐4	☐5	☐9

SECTION G: **Your Medical Office**

How much do you agree or disagree with the following statements?	Strongly Disagree	Disagree	Neither Agree nor Disagree	Agree	Strongly Agree	Does Not Apply or Don't Know
53. When there is a problem in our Center, we see if we need to change the way we do things	☐1	☐2	☐3	☐4	☐5	☐9

How much do you agree or disagree with the following statements?	Strongly Disagree	Disagree	Neither Agree nor Disagree	Agree	Strongly Agree	Does Not Apply or Don't Know
54. Our Center processes are good at preventing mistakes that could affect patients	☐1	☐2	☐3	☐4	☐5	☐9
55. Mistakes happen more than they should in this Center	☐1	☐2	☐3	☐4	☐5	☐9
56. It is just by chance that we don't make more mistakes that affect our patients	☐1	☐2	☐3	☐4	☐5	☐9
57. This Center is good at changing Center processes to make sure the same problems don't happen again	☐1	☐2	☐3	☐4	☐5	☐9
58. In this Center, getting more work done is more important than quality of care	☐1	☐2	☐3	☐4	☐5	☐9
59. After this Center makes changes to improve the patient care process, we check to see if the changes worked	☐1	☐2	☐3	☐4	☐5	☐9

SECTION H: **Overall Ratings**

Overall Ratings on Quality

Overall, how would you rate your Primary Care Center on each the following areas of health care quality?		Poor	Fair	Good	Very good	Excellent
**60. Patient centered**	Is responsive to individual patient preferences, needs, and values	☐1	☐2	☐3	☐4	☐5
**61. Effective**	Is based on scientific knowledge	☐1	☐2	☐3	☐4	☐5
**62. Timely**	Minimizes waits and potentially harmful delays	☐1	☐2	☐3	☐4	☐5
**63. Efficient**	Ensures cost-effective care (avoids waste, overuse, and misuse of services)	☐1	☐2	☐3	☐4	☐5
**64. Equitable**	Provides the same quality of care to all individuals regardless of gender, race, ethnicity, socioeconomic status, language, etc.	☐1	☐2	☐3	☐4	☐5

Overall Rating on Patient Safety

	Poor	Fair	Good	Very good	Excellent
**65. Overall, how would you rate the systems and clinical processes your Primary Care Center has in place to prevent, catch, and correct problems that have the potential to affect patients?**	☐1	☐2	☐3	☐4	☐5

SECTION I: **Background Questions**

66. How long have you worked in <u>this Primary Care Center location</u>?

- 1. Less than 2 months
- 2. 2 months to less than 1 year
- 3. 1 year to less than 3 years
- 4. 3 years to less than 6 years
- 5. 6 years to less than 11 years
- 6. 11 years or more

67. Typically, how many hours per week do you work in <u>this Primary Care Center location</u>?

- 1. 1 to 4 hours per week
- 2. 5 to 16 hours per week
- 3. 17 to 24 hours per week
- 4. 25 to 32 hours per week
- 5. 33 to 40 hours per week
- 6. 41 hours per week or more

68. What is your job title in this Center? Check ONE category that best applies to your job.

- 1. **Primary Care Center Head**
- 2. **Trustee / Administrative Supervisor**
- 3. **Receptionist / Medical Records**
- 4. **Information Systems Officer / Secretariat**
- 5. **Hotel Services Supervisor / Head of Cleaners**

Nurse

- 6. Assistant Nurse
- 7. Nurse, Senior Nurse
- 8. Head Nurse

General Practitioner

- 9. General Practitioner
- 10. Senior General Practitioner (A/B)

Family medicine

- 11. Assistant Register/Register
- 12. Senior Register/ Specialist/ Senior Specialist/ Consultant

Physician (Other Specialties)

- 13. Assistant Register/Register
- 14. Senior Register/ Specialist/ Senior Specialist/ Consultant

Health Inspector

- 15. Health Inspector / Senior Health Inspector
- 16. Health Supervisor / Assistant Head; Supervisors
- 17. Head of Health Supervisors

Pharmacist:

- 18. Junior Pharmacist / Pharmacist
- 19. Senior Pharmacist / Specialist / Senior Specialist
- 20. Head of Pharmacy

Laboratory

- 21. Practitioner / Senior Practitioner
- 22. Specialist / Senior Specialist /
- 23. Head Specialists

- 24. **Technicians** (Lab., Pharmacy, Dental, Nursing, X- Ray, Etc.)
- 25. **Phlebotomists**
- 26. **Other Position; Please specify**: ____

69. **What is your department in <u>this Primary Care Center</u>? <u>Check ONE category that best applies</u>.**

- 1. **<u>Primary Care Clinics</u>:** General medicine clinics, diabetes, chronic diseases and other specialized care clinics
- 2. **Specialized clinics from the hospitals**
- 3. **Preventive Health**
- 4. **Elderly Health Clinics**
- 5. **School Health Clinics**
- 6. **Dental Department**
- 7. **Nursing Department**
- 8. **Pharmacy Department**
- 9. **Laboratory Department**
- 10. **Radiology Department / Mammography / Dental Radiology**
- 11. **Administrative Departments**
- 12. **Reception and Medical Records**
- 13. **Hotel services**
- 14. **Other; Please specify**: ______________________________

70. **What is your highest educational credential:**

- 1. **less than high school**
- 2. **High school**
- 3. **Technical school**
- 4. **University degree**
- 5. **Master**
- 6. **PhD/Board certified**
- 7. **Fellowship degree**
- 8. **Other; Please specify:** ______________________________

71. **Have you ever attended any courses/lectures about patient safety:**

- 1. **Yes**
  - Mention number of hours attended ______________________
  - Year of these course/lectures ______________________
- 2. **No**

72. **Gender:**

- 1. Male
- 2. Female

73. **Age**:

- 1. Below 30 years
- 2. 30 - 45 years
- 3. 46 - 55 years
- 4. Over 55 years

74. **Nationality:**

- 1. Kuwaiti
- 2. Arabian
- 3. Asian
- 4. European/ American
- 5. Other; Please specify: ______________________

SECTION J: **Comments and Suggestions**

75. **Please feel free to write any comments you may have about patient safety or quality of care in your Primary Care Center.**

THANK YOU FOR COMPLETING THIS SURVEY.
