# Supplementary material for: Baseline assessment of patient safety culture in primary care centres in Kuwait: a national cross-sectional study
Source: BMC Health Serv Res. 2021 Oct 28;21:1172. doi: 10.1186/s12913-021-07199-1 (PMC8555195; doi:10.1186/s12913-021-07199-1)
Supplement: Supplementary file 2 — Additional file 2. [file 12913_2021_7199_MOESM2_ESM.pdf]

الرمز:

دراسة سلامة المرضى بالمركز الصحي

تعليمات تعبئة الاستبيان

يعنى هذا الاستبيان بطرق العمل بالمركز الصحي الذي تعمل به، وتحديداً المسائل التي تتعلق بشكل عام بسلامة المرضى وجودة الرعاية الصحية التي يتم تقديمها للمرضى في المركز.

في هذه الاستبيان، يقصد بمصطلح **مقدمي الرعاية الطبية** أفراد الرعاية الطبية من الأطباء والممرضين ممن لهم دور في تشخيص المرضى وعلاجهم وتوصيف الأدوية. أما مصطلح **الموظفين** فنعني به جميع الأفراد الآخرين ممن يعملون في المركز.

- في حال كانت أحد الأسئلة لا تنطبق عليك أو كنت لا تعرف الإجابة، نرجو اختيار إجابة "**لا ينطبق أو لا أعلم**".
- في حال كنت تعمل في أكثر من مركز صحي، نرجو أن تكون إجابتك عن المركز الذي تلقيّت فيه هذا الاستبيان – وألّا تكون حول ممارستك للمهنة بشكل عام.
- في حال كان المركز الصحي الذي تعمل به ضمن مجموعة من المراكز الصحية الأخرى، نرجو أن تركّز إجاباتك على المركز المحدّد الذي تنتسب إليه.

الفقرة (أ): المسائل التي تتعلق بسلامة المرضى وجودة الرعاية

القائمة التالية تبيّن بعض المسائل التي قد تحدث في المراكز الصحية، مما قد تؤثر على سلامة المرضى وجودة الرعاية.

بتقديرك، ما هي وتيرة حدوث (مدى تكرار) مثل هذه المسائل في المركز الصحي حيث تعمل على مدار الأشهر 12 الماضية؟

	يومياً	أسبوعياً	شهرياً	عدة مرات في الأشهر 12 الماضية	مرة أو مرتين في الأشهر 12 الماضية	لم يحدث في الأشهر 12 الماضية	لا ينطبق أو لا أعلم
**الحصول على الرعاية**							
**1.** لم يتمكن أحد المرضى من الحصول على موعد خلال أول 48 ساعة بالرغم من حالته الحرجة / العاجلة	☐1	☐2	☐3	☐4	☐5	☐6	☐9
**التعرّف على المرضى**							
**2.** تمّ استخدام سجّلات طبية خاطئة للمريض	☐1	☐2	☐3	☐4	☐5	☐6	☐9
**السجّلات الطبية**							
**3.** لم يتوفر الملف الطبي للمريض عند الحاجة إليه	☐1	☐2	☐3	☐4	☐5	☐6	☐9
**4.** تم إدخال معلومات/بيانات (سواء إدخال مباشر أو مسح ضوئي) طبية في السجل الطبي الخطأ.	☐1	☐2	☐3	☐4	☐5	☐6	☐9
**المعدّات الطبية**							
**5.** لم تكن المعدّات الطبية تعمل بشكل سليم أو كانت تحتاج إلى تصليح أو استبدال.	☐1	☐2	☐3	☐4	☐5	☐6	☐9

	يومياً	أسبوعياً	شهرياً	عدة مرات فى الأشهر 12 الماضية	مرة أو مرتين فى الأشهر 12 الماضية	لم يحدث فى الأشهر 12 الماضية	لا ينطبق أو لا أعلم
**الأدوية**							
**6.** اتصلت صيدلية، بمركزنا الصحي للاستيضاح عن إحدى الوصفات الطبية أو تصحيحها.	☐1	☐2	☐3	☐4	☐5	☐6	☐9
**7.** لم يتم تحديث قائمة الأدوية لمريض ما أثناء زيارته.	☐1	☐2	☐3	☐4	☐5	☐6	☐9
**الفحوصات التشخيصية**							
**8.** لم تتوفر نتائج فحوصات مخبرية أو صور تشخيصية عند الحاجة إليها.	☐1	☐2	☐3	☐4	☐5	☐6	☐9
**9.** لم تتم متابعة نتيجة فحص مخبري أو صورة تشخيصية حرجة (critical) غير اعتيادية (abnormal)، خلال يوم عمل واحد.	☐1	☐2	☐3	☐4	☐5	☐6	☐9

الفقرة (ب): تبادل المعلومات داخل المركز

على مدار الأشهر 12 الماضية، ما هي وتيرة حدوث (مدى تكرار) المشاكل التي واجهتها على صعيد تبادل المعلومات الدقيقة والكاملة في الوقت المناسب، مع كل من:

	مشاكل يومياً	مشاكل أسبوعياً	مشاكل شهرياً	مشاكل مرات معدودة فى الأشهر 12 الماضية	مشاكل مرة أو مرتين فى الأشهر 12 الماضية	لم يحدث فى الأشهر 12 الماضية	لا ينطبق أو لا أعلم
**10.** خدمات الفحوصات المخبرية داخل المركز؟	☐1	☐2	☐3	☐4	☐5	☐6	☐9
**11.** خدمات التصوير التشخيصي داخل المركز؟	☐1	☐2	☐3	☐4	☐5	☐6	☐9
**12.** الأطباء /العيادات الأخرى؟	☐1	☐2	☐3	☐4	☐5	☐6	☐9
**13.** صيدلية المركز؟	☐1	☐2	☐3	☐4	☐5	☐6	☐9
الخدمات الأخرى داخل المركز؟ (يرجى التحديد): **14.**	☐1	☐2	☐3	☐4	☐5	☐6	☐9
**15.**	☐1	☐2	☐3	☐4	☐5	☐6	☐9

الفقرة (ج): تبادل المعلومات مع الجهات الخارجية

على مدار الأشهر 12 الماضية، ما هي وتيرة حدوث (مدى تكرار) المشاكل التي واجهها مركزكم على صعيد تبادل المعلومات الدقيقة والكاملة في الوقت المناسب، مع كل من:

	مشاكل يومياً	مشاكل أسبوعياً	مشاكل شهرياً	مشاكل مرات معدودة فى الأشهر 12 الماضية	مشاكل مرة أو مرتين فى الأشهر 12 الماضية	لم يحدث فى الأشهر 12 الماضية	لا ينطبق أو لا أعلم
**16.** مراكز الفحوصات المخبرية الخارجية؟	☐1	☐2	☐3	☐4	☐5	☐6	☐9
**17.** مراكز التصوير التشخيصي الخارجية؟	☐1	☐2	☐3	☐4	☐5	☐6	☐9
**18.** الصيدليات الخارجية؟	☐1	☐2	☐3	☐4	☐5	☐6	☐9
**19.** المستشفيات؟	☐1	☐2	☐3	☐4	☐5	☐6	☐9
الجهات الخارجية الأخرى؟ (يرجى التحديد): **.20**	☐1	☐2	☐3	☐4	☐5	☐6	☐9
**.21**	☐1	☐2	☐3	☐4	☐5	☐6	☐9

الفقرة (د): العمل في مركزكم الصحي

**إلى أي مدى توافق أو تعترض على العبارات التالية؟**	أعترض بشدة	أعترض	لا أوافق ولا أعترض (حيادي)	أوافق	أوافق بشدة	لا ينطبق أو لا أعلم
**22.** عندما ينشغل أحد العاملين في المركز إلى حدّ كبير، يقدّم الآخرون له المساعدة.	☐1	☐2	☐3	☐4	☐5	☐9
**23.** في هذا المركز، هناك علاقة عمل جيدة بين مقدمي الرعاية الطبية (الأطباء، الممرضين) والموظفين (جميع الأفراد الآخرين ممن يعملون في المركز).	☐1	☐2	☐3	☐4	☐5	☐9
**24.** في هذا المركز، نشعر غالباً بالضغط للإسراع خلال تقديم خدمات الرعاية للمرضى.	☐1	☐2	☐3	☐4	☐5	☐9
**25.** هذا المركز يدرب الموظفين عند إدخال آليات عمل وإجراءات جديدة.	☐1	☐2	☐3	☐4	☐5	☐9
**26.** في هذا المركز، نعامل بعضنا البعض باحترام.	☐1	☐2	☐3	☐4	☐5	☐9
**27.** في هذا المركز لدينا الكثير من المرضى بالنسبة لعدد مقدمي الرعاية الطبية.	☐1	☐2	☐3	☐4	☐5	☐9

إلى أي مدى توافق أو تعترض على العبارات التالية؟	أعترض بشدة	أعترض	لا أوافق ولا أعترض (حيادي)	أوافق	أوافق بشدة	لا ينطبق أو لا أعلم
**28.** يحرص المركز على أن يحصل الموظفون على التدريب العملي الذي يحتاجونه للوظيفة.	☐1	☐2	☐3	☐4	☐5	☐9
**29.** هذا المركز غير منظم بالدرجة التي يجب أن يكون عليها من التنظيم.	☐1	☐2	☐3	☐4	☐5	☐9
**30.** لدينا إجراءات جيّدة للتحقق من سير العمل بالشكل الصحيح في هذا المركز.	☐1	☐2	☐3	☐4	☐5	☐9
**31.** يُطلَبُ من الموظفين في هذا المركز أداء مهام لم يتم تدريبهم عليها.	☐1	☐2	☐3	☐4	☐5	☐9
**32.** لدينا عدد كافٍ من الموظفين للتعامل مع عدد المرضى.	☐1	☐2	☐3	☐4	☐5	☐9
**33.** لدينا مشاكل فيما يتعلق بسير العمل في هذا المركز.	☐1	☐2	☐3	☐4	☐5	☐9
**34.** هذا المركز يؤكد على العمل بروح الفريق في رعاية المرضى.	☐1	☐2	☐3	☐4	☐5	☐9
**35.** في هذا المركز الكثير من المرضى، مما يجعلنا غير قادرين على إنجاز جميع الأمور بكفاءة.	☐1	☐2	☐3	☐4	☐5	☐9
**36.** يتّبع الموظفين في هذا المركز آليات عمل موحدة لأداء المهام.	☐1	☐2	☐3	☐4	☐5	☐9

الفقرة (هـ): التواصل والمتابعة

ما مدى تكرار الأمور التالية في مركزك الصحي؟	أبداً	نادراً	أحياناً	معظم الوقت	دائماً	لا ينطبق أو لا أعلم
**37.** إن مقدمي الرعاية الطبية في هذا المركز منفتحين لأفكار الموظفين حول كيفية تحسين آليات العمل.	☐1	☐2	☐3	☐4	☐5	☐9
**38.** يتم تشجيع الموظفين في هذا المركز على التعبير عن وجهات نظر بديلة.	☐1	☐2	☐3	☐4	☐5	☐9
**39.** يقوم المركز بتذكير المرضى عند حاجتهم لأخذ موعد للمعاينة الوقائية أو الروتينية.	☐1	☐2	☐3	☐4	☐5	☐9
**40.** يخشى الموظفون في المركز من طرح أسئلة عندما يبدو أن الأمور لا تسير على ما يرام.	☐1	☐2	☐3	☐4	☐5	☐9
**41.** هذا المركز يوثق مدي التزام مرضى الرعاية المزمنة لخطط العلاج الخاصة بهم.	☐1	☐2	☐3	☐4	☐5	☐9
**42.** يقوم مركزنا بالمتابعة في حال عدم تلقي تقرير ننتظره من مقدم خدمة خارجي.	☐1	☐2	☐3	☐4	☐5	☐9
**43.** يشعر الموظفون بأن أخطاءهم يتم احتسابها عليهم.	☐1	☐2	☐3	☐4	☐5	☐9

ما مدى تكرار الأمور التالية في مركزك الصحي؟	أبداً	نادراً	أحياناً	معظم الوقت	دائماً	لا ينطبق أو لا أعلم
**44.** يجري الحديث بصراحة (انفتاح) بين مقدمي الرعاية الطبية والموظفين، فيما يتعلق بمشاكل المركز.	☐1	☐2	☐3	☐4	☐5	☐9
**45.** يقوم المركز بمتابعة المرضى الذين يحتاجون إلى متابعة.	☐1	☐2	☐3	☐4	☐5	☐9
**46.** في هذا المركز، من الصعب التحدث بصراحة في حالات الاعتراض.	☐1	☐2	☐3	☐4	☐5	☐9
**47.** في هذا المركز، تتم مناقشة الآليات الممكنة لتجنّب الأخطاء قبل تكرارها.	☐1	☐2	☐3	☐4	☐5	☐9
**48.** يبدي موظفو المركز ميلاً للإبلاغ عن الأخطاء التي يلاحظونها.	☐1	☐2	☐3	☐4	☐5	☐9

الفقرة (و): الدعم من الجهة المالكة/ الشريك الإداري / القيادة

أ. هل أنت جهة مالكة أو شريكاً إدارياً، أو في موقع قيادي تتضمن مسؤولياته اتخاذ قرارات مالية فيما يخص المركز الصحي؟

☐1 نعم ← انتقل إلى الفقرة (ز)

☐2 كلا ← تابع الفقرة (و) أدناه

إلى أي مدى توافق أو تعترض على العبارات التالية فيما يخص الجهات المالكة / الشركاء الإداريين / القيادة في مركزك الصحي؟	أعترض بشدة	أعترض	لا أوافق ولا أعترض (حيادي)	أوافق	أوافق بشدة	لا ينطبق أو لا أعلم
**49.** لا يستثمرون ما يكفي من الموارد لتحسين جودة الرعاية في هذا المركز.	☐1	☐2	☐3	☐4	☐5	☐9
**50.** يتغاضون عن أخطاء رعاية المرضى التي تحدث مراراً وتكراراً.	☐1	☐2	☐3	☐4	☐5	☐9
**51.** يعطون أولوية كبيرة لإجراءات تحسين رعاية المرضى.	☐1	☐2	☐3	☐4	☐5	☐9
**52.** غالباً ما يتخذون القرارات بناءً على ما هو الأفضل للمركز الصحي، وليس ما هو الأفضل للمرضى.	☐1	☐2	☐3	☐4	☐5	☐9

الفقرة (ز): في المركز الصحي الذي يخصّك

إلى أي مدى توافق أو تعترض على العبارات التالية؟	أعترض بشدة	أعترض	لا أوافق أعترض ولا (حيادي)	أوافق	أوافق بشدة	لا ينطبق أو لا أعلم
**53.** عندما تحدث مشكلة في مركزنا، نحن نبحث عما إذا كانت هناك الحاجة إلى تغيير طريقة عملنا.	☐1	☐2	☐3	☐4	☐5	☐9
**54.** لدينا في المركز آليات عمل جيدة لمنع الأخطاء التي قد تؤثر على المرضى.	☐1	☐2	☐3	☐4	☐5	☐9

إلى أي مدى توافق أو تعترض على العبارات التالية؟	أعترض بشدة	أعترض	لا أوافق أعترض ولا (حيادي)	أوافق	أوافق بشدة	لا ينطبق أو لا أعلم
**55.** تحدث الأخطاء أكثر مما يجب في هذا المركز.	☐1	☐2	☐3	☐4	☐5	☐9
**56.** من باب الصدفة فقط، اننا لا نرتكب مزيد من الأخطاء التي تؤثر على مرضانا.	☐1	☐2	☐3	☐4	☐5	☐9
**57.** يتميز هذا المركز الصحي بالقدرة على تغيير آليات العمل لضمان عدم تكرّر الأخطاء نفسها مجدداً.	☐1	☐2	☐3	☐4	☐5	☐9
**58.** في هذا المركز الصحي، القيام بالمزيد من العمل هو أكثر أهمية من جودة الرعاية.	☐1	☐2	☐3	☐4	☐5	☐9
**59.** بعد استحداث تغييرات في المركز الصحي لتحسين آليات عمل رعاية المريض، نحن نتحقق من أن هذه التغييرات تؤدي غرضها.	☐1	☐2	☐3	☐4	☐5	☐9

الفقرة (ح): التقييم الإجمالي

التقييم الإجمالي بخصوص الجودة

بشكل عام، كيف تقيّم الجوانب التالية بمركزك في فيما يتعلق بجودة الرعاية الصحية؟		ضعيف	مقبول	جيد	جيد جداً	ممتاز
**60. الرعاية المتمركزة على المريض**	يتجاوب مع التفضيلات الفردية لكل مريض، واحتياجاته، وقيمه.	☐1	☐2	☐3	☐4	☐5
**61. فعّال**	يعتمد على المعرفة العلمية	☐1	☐2	☐3	☐4	☐5
**62. تقديم الرعاية في الوقت المناسب**	يقلص فترات الانتظار والتأخير الذي قد يؤدي إلى ضرر	☐1	☐2	☐3	☐4	☐5
**63. الاستخدام الأمثل**	يحرص على فعالية التكلفة في الرعاية (تجنّب الهدر والمبالغة بالاستخدام وإساءة استخدام الخدمات)	☐1	☐2	☐3	☐4	☐5
**64. المساواة**	يوفر نفس مستوى جودة الرعاية لجميع الأفراد، بغض النظر عن الجنس أو العرق أو الدين أو المستوي الاقتصادي والاجتماعي أو اللغة، الخ ...	☐1	☐2	☐3	☐4	☐5

التقييم الإجمالي لسلامة المرضى

**65.** بشكل عام، كيف تقيم الأنظمة والإجراءات الطبية في مركزك الطبي لمنع، وتصحيح المشاكل التي يمكن أن تؤثر على المرضى؟	ضعيف	مقبول	جيد	جيد جداً	ممتاز
	☐1	☐2	☐3	☐4	☐5

الفقرة (ط): بيانات شخصية

66. كم مضى على عملك في <u>هذا المركز الصحي تحديداً</u>؟

1. أقل من شهرين
2. من شهرين إلى أقل من سنة واحدة
3. من سنة واحدة إلى أقل من 3 سنوات
4. من 3 سنوات إلى أقل من 6 سنوات
5. من 6 سنوات إلى أقل من 11 سنة
6. 11 سنة أو أكثر

67. عادةً، ما هو عدد الساعات التي تعمل بها أسبوعياً في <u>هذا المركز الصحي تحديداً</u>؟

1. 1–4 ساعات أسبوعياً
2. 5–16 ساعة أسبوعياً
3. 17–24 ساعة أسبوعياً
4. 25 – 32 ساعة أسبوعياً
5. 33 – 40 ساعة أسبوعياً
6. 41 ساعة أسبوعياً أو أكثر

68. ما هو مسماك الوظيفي؟ اختر الإجابة التي تجدها أكثر ملائمة (الرجاء اختيار إجابة واحدة فقط)

1. **رئيس المركز**
2. **أمين المركز/ المشرف الإداري**
3. **موظفي الاستقبال/ سجلات طبية**
4. **موظف نظم المعلومات/ سكرتارية**
5. **مشرف خدمات فندقية / رئيس عمال النظافة**

ممرض

6. ممرض مساعد
7. ممرض، ممرض أول
8. مسؤول قسم تمريض

ممارس عام

9. ممارس عام
10. ممارس عام أول (أ / ب)

طبيب عائلة

11. مساعد مسجل، مسجل
12. مسجل أول/ اختصاصي/ اختصاصي أول / استشاري

طبيب (تخصصات أخرى)

13. مساعد مسجل، مسجل
14. مسجل أول/ اختصاصي/ اختصاصي أول / استشاري

مفتش صحي

15. مفتش صحي / مفتش صحي أول
16. مشرف صحي / مساعد رئيس مشرفين
17. رئيس مشرفين صحين

صيدلي

18. صيدلي مبتدأ، صيدلي
19. صيدلي أول، اختصاصي، اختصاصي أول
20. رئيس صيادلة

أخصائي مختبر

21. ممارس، ممارس أول
22. اختصاصي، اختصاصي أول،
23. رئيس اختصاصيين
24. **فني** (مختبر، صيدلة، أسنان، تمريض، الأشعة، الخ)
25. **فصدة** (phlebotomist)
26. **أخرى، يرجى التحديد:** ....

69.ما هو القسم الخاصة بكم في هذا المركز الصحي؟ (الرجاء اختيار إجابة واحدة فقط)

1. عيادات الطب العام، السكر، الأمراض المزمنة، العيادات التخصصية **التابعة للرعاية الأولية**
2. العيادات التخصصية **التابعة للمستشفيات**
3. الصحة الوقائية
4. عيادات صحة كبار السن
5. الصحة المدرسية
6. قسم الأسنان
7. قسم التمريض
8. قسم الصيدلية
9. قسم المختبر
10. قسم الأشعة / أشعة الثدي/ أشعة الأسنان
11. الأقسام الإدارية
12. موظفي الاستقبال والسجلات طبية
13. الخدمات فندقية
14. **أخرى، رجاء التحديد** ....

70.المستوى الدراسي: ما هي أعلى شهادة/ مؤهل حصلت عليه؟

1. أقل من ثانوي
2. ثانوي
3. ثانوي فني
4. جامعي
5. ماجستير
6. دكتوراه
7. درجة الزمالة
8. **درجة أخرى: حدد**.......................

71.هل حضرت دورات / محاضرات تدريبية عن سلامة المرضى:

1. **نعم**

اذكر عدد الساعات التي حضرتها ----------- ساعة

اذكر العام الذي حضرت فيه الدورات/ المحاضرات التدريبية ----------------------

2. **لا**

72.الجنس: 1. **ذكر** 2. **أنثى**

73.العمر:

1. اقل من 30
2. 30 – 45 عاماً
3. 46 – 55 عاماً
4. أكثر من 55 عاماً

74.الجنسية:

1. كويتي
2. عربي
3. آسيوي
4. اوروبي / امريكي
5. أخرى، رجاء التحديد ....

الفقرة (ط): تعليقات ومقترحات

75.الرجاء كتابة أي ملاحظات أو تعليقات لديكم فيما يتعلق بسلامة المرضى و/ أو جودة الرعاية الصحية في المركز الصحي لديكم.

شكراً لكم على حسن تعاونكم ومشاركتكم القيّمة
